# Distinct Inflammatory and Dissemination Signatures Defined by Macrophage Migration Inhibitory Factor (MIF), Interleukin-8 (IL-8/CXCL8), and Stem Cell Factor (SCF) in Pancreatic Adenocarcinoma

**DOI:** 10.3390/diagnostics16091373

**Published:** 2026-04-30

**Authors:** Augustin Catalin Dima, Daniel Vasile Balaban, Iulia-Ioana Stanescu-Spinu, Ana Teodorescu, George Manucu, Laura Ioana Coman, Alina Dima, Cezar Betianu, Mihai Tanase, Daniela Miricescu, Mariana Jinga, Catalin Carstoiu

**Affiliations:** 1 Clinical Department 14, Faculty of Medicine, Carol Davila University of Medicine and Pharmacy, 050474 Bucharest, Romania; augustin.dima@drd.umfcd.ro (A.C.D.); catalin.carstoiu@umfcd.ro (C.C.); 2 Department of Surgery 2, Doctor Carol Davila Central Military Emergency University Hospital, 010825 Bucharest, Romania; drmtanase@yahoo.com; 3 Clinical Department 5, Faculty of Medicine, Carol Davila University of Medicine and Pharmacy, 050474 Bucharest, Romania; vasile.balaban@umfcd.ro (D.V.B.); alina.dima@umfcd.ro (A.D.); mariana.jinga@umfcd.ro (M.J.); 4 Department of Gastroenterology, Doctor Carol Davila Central Military Emergency University Hospital, 010825 Bucharest, Romania; ana-maria.teodorescu@rez.umfcd.ro (A.T.); georgemanucu95@yahoo.com (G.M.); laura.coman21@yahoo.com (L.I.C.); 5 Discipline of Physiology, Faculty of Dentistry, Carol Davila University of Medicine and Pharmacy, 050474 Bucharest, Romania; 6 Department of Radiology, Doctor Carol Davila Central Military Emergency University Hospital, 010825 Bucharest, Romania; cezar.betianu@gmail.com; 7 Discipline of Biochemistry, Faculty of Dentistry, Carol Davila University of Medicine and Pharmacy, 050474 Bucharest, Romania; daniela.miricescu@umfcd.ro; 8 Department of Orthopaedics and Traumatology, University Emergency Hospital, 050098 Bucharest, Romania

**Keywords:** pancreatic cancer, biomarker, macrophage migration inhibitory factor, interleukin-8, stem cell factor, inflammation

## Abstract

**Background/Objectives:** Pancreatic adenocarcinoma remains one of the most lethal malignancies, largely due to aggressive biological behavior and limited available insight into biomarker-based prognostic stratification. The aim of our research was to investigate the role of macrophage migration inhibitory factors (MIFs), interleukin-8 (IL-8/CXCL8), and stem cell factors (SCFs) in pancreatic adenocarcinoma. **Methods:** In this single-center study, sixty hospitalized patients diagnosed with pancreatic adenocarcinoma were prospectively enrolled, and a cross-sectional analysis of baseline cytokine levels was performed. Serum MIF, IL-8/CXCL8, and SCF were assessed in a single analytical run using Luminex xMAP technology. **Results:** Elevated MIF and IL-8/CXCL8 levels characterized an inflammatory phenotype, associated with leukocytosis, neutrophilia, increased fibrinogen levels, and unequal prevalence of new-onset diabetes. Higher MIF levels were further associated with larger tumor dimension, while IL-8/CXCL8 was associated with increased bilirubin level and recent weight loss (*p* < 0.05). In contrast, increased SCF predicted a dissemination phenotype as defined by metastasis occurrence (65.4% vs. 28.6%, *p* = 0.012). SCF demonstrated significant discriminatory ability for metastasis (AUC 0.712, *p* = 0.013) and remained significantly associated in multivariable analysis. **Conclusions:** MIF and IL-8/CXCL8 primarily reflect inflammation-driven processes, whereas SCF identifies a dissemination-dominant phenotype, suggesting distinct biological pathways underlying disease progression in pancreatic cancer.

## 1. Introduction

Neoplasia remains one of the leading causes of mortality worldwide [1]. While globally, pancreatic cancer is the seventh leading cause of cancer death, in high-income countries, it ranks third after lung and colorectal cancer [2]. Although impressive advances in cancer therapy have been made and the overall cancer mortality has continued to decline throughout 2022–2025 [1], pancreatic cancer maintains one of the least favorable prognoses [1,3], with a 5-year survival rate of only 8–13% [2]. North America, Europe, and Australia have the highest rates of pancreatic cancer [4]. In Europe, the risk of pancreatic cancer occurrence is about 7–12 cases per 100,000 inhabitants [5]. Notwithstanding that, the incidence rates have shown increasing trends of 1.0% per year [2] and global analysis indicates that by 2030, the pancreatic cancer incidence is expected to show a slow but steady increase [3].

Non-modifiable risk factors for pancreatic cancer include age, sex, blood type, location, family history, genetic susceptibility, and diabetes. Modifiable risk factors include gut microflora, alcohol use, chronic pancreatitis, smoking, obesity, diet, and infections [6].

Patients with familial risk factors are nine times more likely to develop pancreatic cancer, and the risk rises to thirty-two times higher if three or more first-degree relatives have been diagnosed [7]. Studies have indicated that individuals with blood types A, B, or AB face a higher risk compared to those with type O. This can be attributed to changes in blood-type antigens that may interfere with cell signaling, adhesion, and the immune system’s capacity to eliminate preneoplastic cells [8]. A characteristic feature of pancreatic cancer is the high frequency of oncogenic mutations in the *KRAS* gene. The *KRAS* oncogene is essential for the initiation and continued growth of pancreatic tumors, and its signaling pathway is a primary target for therapy [9]. Besides *KRAS*, point mutations involving *CDKN2A* (p16), *TP53*, *SMAD4*, *BRCA2*, *BRCA1*, *STK11*, *PRSS1*, and *MMR25* are among the most commonly involved in pancreatic cancer development [6].

Cigarette smoking is a well-known risk factor for pancreatic cancer, linked to DNA damage, inflammation, and fibrosis [10]. On the other hand, it was demonstrated that gut bacteria can migrate to the pancreas, indicating the potential for direct interaction between gut bacteria and the pancreatic microenvironment [11]. Furthermore, studies showed that lower levels of *Neisseria elongata* and *Streptococcus mitis*, along with higher levels of *Porphyromonas gingivalis* and *Granulicatella adiacens,* are associated with an increased risk of pancreatic cancer [12].

Furthermore, the consumption of red, unprocessed meats and saturated fats is associated with an increased risk of pancreatic cancer, especially after the age of 50 years [13]. Also, previous research shows that about 80% of people with pancreatic cancer also have glucose intolerance or diabetes. Hyperinsulinemia, specific to type 2 diabetes, and high insulin-like growth factor-1 levels can induce pancreatic glandular proliferation and specific cellular interactions, leading to fibrosis, cellular proliferation, and inhibition of apoptosis. These processes are responsible for the desmoplastic reaction and hyperplasia frequently observed in pancreatic neoplastic tissues [14].

Moreover, excessive alcohol consumption (usually defined as more than three drinks per day) has been associated with a slight increase in pancreatic ductal adenocarcinoma risk. Chronic pancreatitis, often linked to alcohol use and smoking, presents a substantially higher risk of developing pancreatic ductal adenocarcinoma [15].

**Figure 1:**
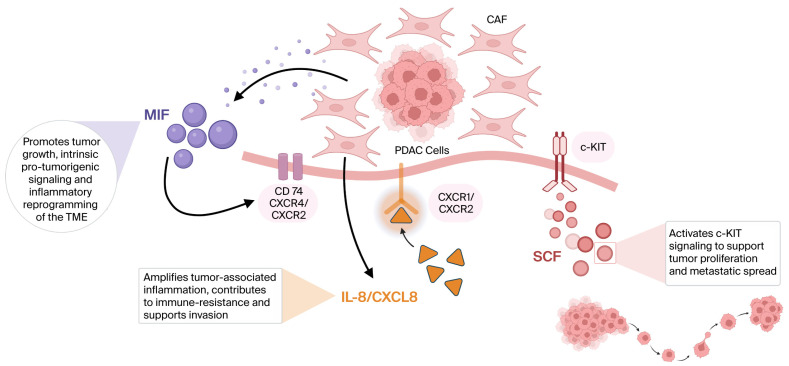
Crosstalk between PDAC biomarkers and the tumor microenvironment, and inflammation and dissemination pathways: MIF and IL-8 predominantly reflect an inflammatory tumor–stroma milieu, whereas SCF engages c-KIT-dependent signaling associated with tumor proliferation and metastatic dissemination. Abbreviations: MIF—macrophage migration inhibitory factor, PDAC—pancreatic ductal adenocarcinoma, CAF—cancer-associated fibroblast, TME—tumor microenvironment, SCF—stem cell factor, IL-8—interleukin 8. Created with BioRender.com (2026).

These factors are linked to immune dysfunction and the initiation and maintenance of an inflammatory microenvironment within the pancreas, thus contributing to the overproduction of cytokines, chemokines, and growth factors. [16,17]. Tumor behavior results from complex interactions, malignant potential, and disease aggressiveness that involve tumoral cells and also the immune and tumor microenvironment [18,19]. Inflammatory cytokines within the tumor microenvironment trigger complex interactions involved in tumor progression, angiogenesis, and resistance to anti-tumoral immune responses (Figure 1) [18,19].

In view of these observations, biomarker-based research capturing the temporal disease dynamics might provide insights into the evolving biology of the disease [20]. In this context, we analyzed the macrophage migration inhibitory factor (MIF), interleukin-8 (IL-8/CXCL8), and stem cell factor (SCF) serum levels in patients with pancreatic adenocarcinoma.

MIF was first described in 1966 as a T-cell-derived cytokine during delayed-type hypersensitivity [21]; it is a 12.5 kDa protein composed of two antiparallel alpha-helices and six beta pleated sheets [22]. MIF is widely expressed across multiple tissues and displays pleiotropic functions in inflammation, immune reactions, and cancer tumorigenesis [21,22,23,24,25]. MIF functions as a negative regulator of p53-mediated growth arrest and apoptosis [26], acts as a counter-regulator of glucocorticoid-mediated immunosuppression [22], and plays a regulatory role in the IL-2–Treg axis, promoting tumoral growth [27]. Further, MIF exerts regulatory control in inflammatory reactions [28], especially in sepsis [28,29]. MIF stimulates inflammation notably through the induction of other cytokines and contributes to the NLRP3 inflammasome activation [30].

Interleukin-8 (IL-8/CXCL8) is an 8-10 kDa cysteine-X-cysteine (CXC) Motif Chemokine ligand 8 (CXCL8), part of the CXC chemokine group [31,32], including immune and neoplastic tumoral cells [33].

Through autocrine signaling, the IL-8/CXCL8 is involved in promoting tumoral cellular proliferation, migration, and invasion [34]. The pro-inflammatory IL-8/CXCL8, initially molecularly cloned as a neutrophil chemotactic factor [16], is secreted by various cell types and acts as a potent neutrophil-recruiting chemokine in inflammatory processes [33,35].

Findings on the IL-8/CXCL8 higher levels when compared to controls were reported across several tumor types, such as colon, lung, breast, and pancreatic [19,34,36]; however, conclusive data are lacking. Also, CXCR1, a receptor for CXCL8/IL-8, has been shown to be linked to cancer stem cells in certain neoplasia [34].

SCF, also referred to as mast cell growth factor, steel factor, and KIT ligand, is a multifunctional cytokine involved in oncogenic processes initiation and progression [37,38]. SCF is a cytokine that selectively interacts with the tyrosine kinase receptor c-KIT (CD117) [39,40], belonging to the type III receptor tyrosine kinase (RTK) family [36].

Neoplastic tumor cells display an overactivation of c-KIT due to a gain-of-function mutation or receptor overexpression [39]. SCF and its receptor c-KIT are upregulated in various cancers, including gastrointestinal stromal tumors, breast cancer, myeloid leukemia, cutaneous melanoma, and glioma [37,41]. The SCF/c-KIT interaction initiates activation of kinase activity and the onset of several signal transduction pathways involved in cellular proliferation, differentiation, migration, or apoptosis in several tissues [39,42]. The SCF/c-KIT system might play a role in regulating growth in normal pancreatic tissue, a mechanism that is altered during malignant transformation [41].

Despite growing evidence exploring the involvement of MIF, IL-8/CXCL8, and SCF in various cancers (see Figure 1), data focusing on pancreatic cancer remains poorly defined and not fully characterized, highlighting the need for further investigation. Considering all the aforementioned aspects, this study aimed to investigate whether circulating MIF, IL-8/CXCL8, and SCF are associated with tumor-related inflammation or dissemination in pancreatic cancer, thereby providing new insights into the complex mechanisms underlying this malignancy.

## 2. Materials and Methods

### 2.1. Study Population

A hospital-based, single-center cross-sectional study was conducted, with consecutive enrollment of adult patients (older than 18 years) admitted to the Gastroenterology Department of the Central Military Emergency University Hospital, between 1 February and 1 December 2023, who were willing to participate in the research.

Patients with known chronic inflammatory, active infections, or a diagnosis of another concurrent malignancy were excluded. Patients with ongoing immunosuppressive or immunomodulatory therapy were also excluded from analysis.

At baseline, comprehensive clinical characteristics, laboratory data, and imaging findings were prospectively collected and uniformly registered in all cases.

In all cases, standardized study forms were completed at baseline to ensure methodological homogeneity. The diagnosis of pancreatic adenocarcinoma was histologically confirmed. All patients included signed an informed consent and agreed to the study procedures.

Based on contrast-enhanced computed tomography imaging findings, vascular invasion was defined as tumor contact with adjacent major vessels (superior mesenteric artery, celiac axis, common hepatic artery, portal and superior mesenteric veins, while the local invasion was defined as direct tumor extension beyond the pancreas into adjacent structures (peripancreatic fat and mesentery, retroperitoneal tissues, duodenum, stomach, spleen, or adrenal gland). The CT imaging interpretation was performed by the radiologists as part of standard care, according to institutional practice.

Metastatic disease was reported when at least one metastatic location was clearly documented; however, a comprehensive evaluation regarding all metastatic sites was not consistently documented for all patients.

### 2.2. Sample Analysis

For biomarker assessment, peripheral blood samples were collected and processed according to a standardized protocol at baseline in all cases. After centrifugation at 4000 rpm for 15 min, each serum was aliquoted and stored at −70 °C in an ultra-low temperature freezer until analysis.

To ensure analytical consistency, samples were thawed simultaneously and processed in a single analytical run to minimize inter-assay variability. Quantification of circulating serum MIF, IL-8/CXCL8, and SCF was further assessed in all samples.

Reagents, including antibody-immobilized beads, seven standards, two quality controls, and a serum matrix, were prepared following the manufacturer’s recommendations. Serum samples were diluted at a ratio of 1:6 with the serum matrix prior to analysis.

Initially, a 200 μL assay buffer was added to each test tube and consequently incubated on a shaker for 10 min to eliminate residual contents. Following the manufacturer’s instructions, the wells received 25 μL each of assay buffer, standards, or controls as appropriate, while sample wells were loaded with 25 μL of assay buffer and 25 μL of diluted serum. Subsequently, 25 μL of mixed magnetic beads was added to all wells.

Mixed beads were added, and plates were incubated overnight at 4 °C for 18 h. Following incubation, the wells’ content was washed three times, after which 25 μL of detection antibodies was added for one hour at room temperature in a plate shaker. Streptavidin–phycoerythrin incubation was carried out for an additional 30 min.

Fluorescence signals were acquired after the addition of 100 μL sheath fluid, and concentrations were calculated using standard curves generated by serial dilution. Analyses were performed using a multianalyte multiplex platform from the Human Circulating Cancer Biomarker Panel (EMD Millipore Corporation, Billerica, MA, USA; Cat. No. HCCBP1MAG-58K).

Calibration curves were generated from seven serially diluted standards, and assay performance was monitored using two quality controls incorporated into each analytical run. Data acquisition was performed using the Luminex^®^ platform, and analysis was conducted with dedicated software. According to the assay manufacturer’s documentation, the minimum detectable concentrations were 0.3 pg/mL for IL-8, 7.6 pg/mL for MIF, and 2.0 pg/mL for SCF.

Oral and written informed consents were obtained from all patients. The study protocol was reviewed and approved by the local Ethics Committee.

### 2.3. Statistical Analysis

Data distribution was assessed prior to analysis. Continuous variables were described using central tendency and dispersion measures appropriate to their distribution, including mean ± standard deviation (SD) or as median (q25 and q75 percentiles) reported, while the categorical ones were reported as percentages. Between-group comparisons were reported using the chi-square test, Mann–Whitney U test, or Kruskal–Wallis H test as appropriate.

Bivariate correlations were assessed using Spearman’s rho coefficient (r). In the absence of validated thresholds, median values were adopted as cut-off points for all variables analyzed.

A receiver operating characteristic (ROC) curve was employed to evaluate biomarker performance, and the area under the curve (AUC) was assessed.

Binary logistic regression models were subsequently used to evaluate potential predictors of the predefined outcome. Multivariate binary logistic regression analysis was constructed to identify independent predictors of the outcome of interest.

Effect estimates were presented as odds ratios (ORs) with corresponding 95% confidence intervals (CIs). A significant level of 0.05 for a two-sided *p*-value was applied. The statistical analyses were performed using the IBM SPSS Statistics, version 25 (IBM Corp., Armonk, NY, USA).

## 3. Results

### 3.1. General Data

The study included sixty consecutive patients diagnosed with pancreatic cancer, with a median age at baseline of 69.5 (64.2; 76.0) years and a relatively balanced gender distribution (male-to-female ratio of 1:1.3). Current or former smoking status was documented in 39.0% of cases, while alcohol consumption was reported in 26.3%.

A descriptive overview of baseline demographic, clinical, and laboratory characteristics of the study group is presented in Table 1. Dietary habits included meat-rich diets in 35.7%, sweet-rich diets in 28.1%, and high-fat diets in 22.8% of cases.

Weight loss was frequently reported in about three-quarters of patients, with a median declared average of 10.0 (4.0; 15.0) kg. Diabetes mellitus, including both long-standing and newly diagnosed forms, was associated in 42.1% cases.

**Figure 2:**
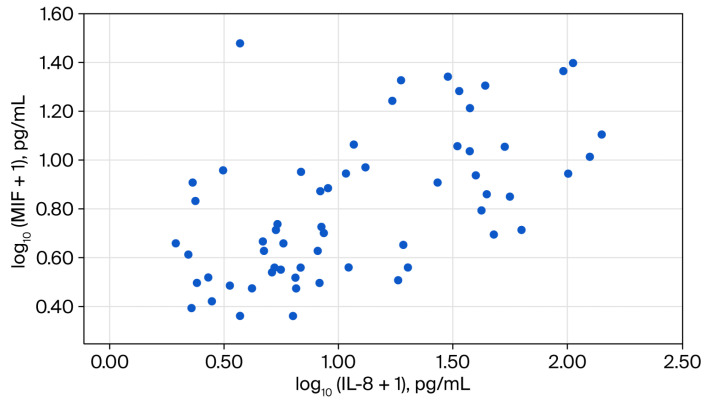
Scatter plot for the association between log-transformed serum values of macrophage migration inhibitory (MIF) and interleukin-8 (IL-8/CXCL8).

**Figure 3:**
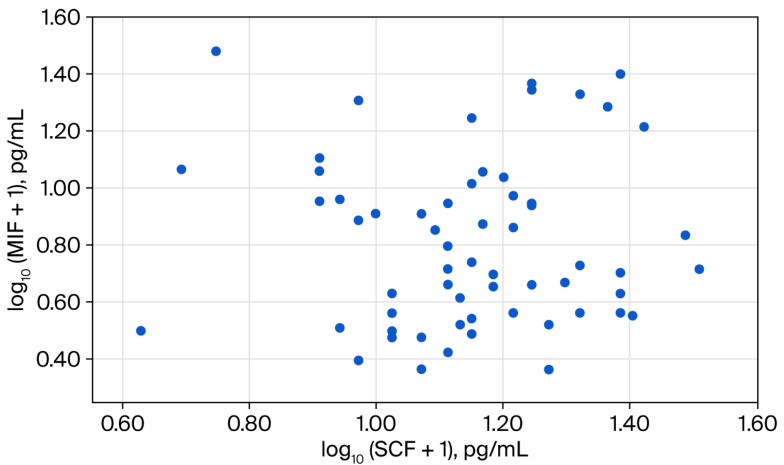
Scatter plot for the association between log-transformed values of serum macrophage migration inhibitory factor (MIF) and stem cell factor (SCF).

**Figure 4:**
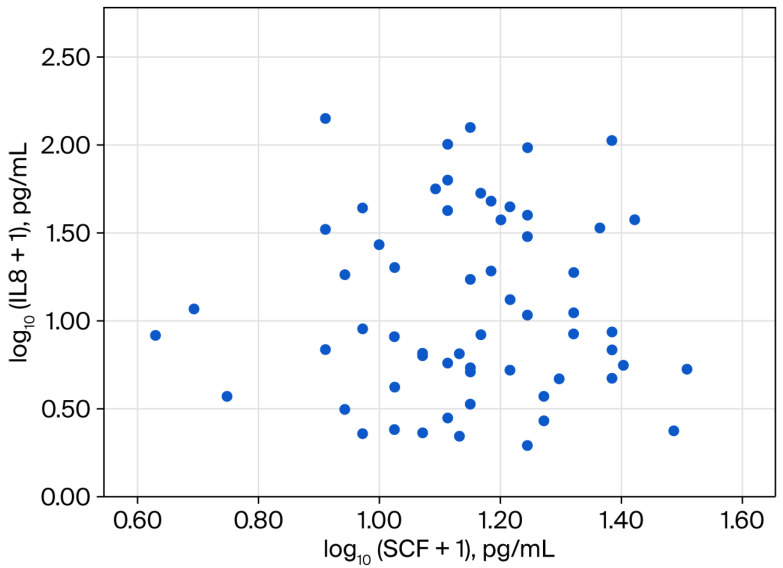
Scatter plot for the association between log-transformed values of serum interleukin-8 (IL-8/CXCL8) and stem cell factor (SCF).

Inflammatory markers showed elevated median values within the study group, with CRP levels of 14.5 mg/L and an ESR of 31.0 mm/h. Serum levels of MIF, IL-8/CXCL8, and SCF showed median (q1; q3) values of 4.4 (2.6; 9.0) pg/mL, 7.5 (3.8–35.5) pg/mL, and 13.1 (9.5–17.4) pg/mL, respectively (correlations presented in Figure 2, Figure 3 and Figure 4).

Tumor-related imaging evaluation indicated local invasion in 87.0% and vascular invasion in 65.9% cases, with metastatic disease present in nearly half of the patients included (see Table 1).

### 3.2. Macrophage Migration Inhibitory Factor (MIF)

Due to the absence of previously established MIF cut-off values in the literature for pancreatic cancer, cases were dichotomized according to the median serum MIF value in both cases and controls tested (5.2 pg/mL) into low versus high MIF groups. The clinical and laboratory baseline characteristics distinguishing between the two groups are detailed in Table 2.

Of note, high MIF levels marked also an increased inflammatory state, with higher serum levels of white blood cells and inflammatory biomarkers, such as leucocytes, neutrophils, fibrinogen, and CRP: 10.2 (8.1–11.7) × 10^9^/L versus 7.2 (5.7–8.5) × 10^9^/L, 6.8 (5.2–8.6) × 10^9^/L versus 4.9 (3.9–5.7) × 10^9^/L, 689.0 (597.2–860.2) mg/dL versus 599.0 (504.0–744.0) mg/dL, and 66.8 (5.8–126.7) mg/dL versus 9.9 (2.6–33.8) mg/dL; however, statistical significance was not achieved for the last parameter mentioned here. Consistently, MIF exhibited significant weakly positive bivariate correlations with CRP (r = 0.356, *p* = 0.015), fibrinogen (r = 0.336, *p* = 0.037), leucocytes (r = 0.345, *p* = 0.009), and neutrophils (r = 0.311, *p* = 0.018), thus supporting the previously observed pattern, as presented in Table 3.

Also, even if a correlation with metastatic disease was not observed, higher MIF levels were more frequently found in cases with larger tumoral dimensions, 34.5% versus 21.1%, *p* = 0.030, see Table 2.

Further, new-onset diabetes mellitus was more commonly reported in patients with pancreatic cancer and higher compared to low MIF levels (27.5% versus 7.1%, *p* = 0.042), as presented in Table 2.

### 3.3. Interleukin-8 (IL-8/CXCL8)

Regarding IL-8/CXCL8, the group-wise comparisons across clinical and laboratory variables showed, similarly to MIF, that elevated IL8/CXCL8 levels were associated with leucocyte and neutrophil counts, as well as higher inflammatory marker levels (ESR, CRP, fibrinogen). Likewise, MIF levels were also significantly associated with the IL-8/CXCL8 levels: 9.5 (5.8–16.9) pg/mL versus 3.1 (2.1–4.8) pg/mL, *p* < 0.001 (see Table 2).

Furthermore, in our research, the elevated IL-8/CXCL8 levels were specifically related to cholestasis as expressed by total and direct serum bilirubin levels: 4.0 (0.8–12.9) versus 0.6 (0.4–4.8) mg/dL, *p* = 0.005, and 2.4 (0.2–7.3) versus 0.15 (0.1–2.7) mg/dL, *p* = 0.009, respectively.

Using Spearman’s rank correlation analysis, IL-8/CXCL8 levels showed a significant positive correlation with fibrinogen (r = 0.403, *p* = 0.011), while correlations with ESR (r = 0.397, *p* = 0.055) and CRP (r = 0.288, *p* = 0.052) did not reach statistical significance, even if a trend toward significance was observed (see Table 3).

In terms of diabetes mellitus status, long-standing diabetes mellitus was more frequent among patients with high IL-8/CXCL8 levels (38.5% vs. 12.9%, *p* = 0.026), in contrast to newly diagnosed diabetes.

In addition, patients who reported recent weight loss at inclusion also had higher IL-8/CXCL8 serum levels compared to those without reported weight loss, 10.6 (5.4; 37.6) versus 4.2 (1.5; 34.7) pg/mL, reaching borderline statistical significance (*p* = 0.050).

### 3.4. Stem Cell Factor (SCF)

In contrast to the other two serum biomarkers analyzed, no significant differences were observed in regard to white blood cell counts or systemic inflammatory markers.

In addition, the metastatic disease was significantly more prevalent among patients with elevated SCF levels: 65.4% versus 28.6%, *p* = 0.012. Notably, among the three biomarkers analyzed, SCF remained the only biomarker associated with the presence of metastasis, both in continuous SCF levels and dichotomized high SCF levels: 15.4 (12.5–19.9) pg/mL versus 11.9 (8.5–15.1) pg/mL, *p* = 0.013 and 73.9% versus 37.5%, *p* = 0.012, respectively (see Supplementary Materials).

### 3.5. Phenotype Stratification

Considering the results presented thus far, patients were stratified into three biological phenotypes based on the cut-off values of MIF, IL-8/CXCL8, and SCF. First, a low inflammatory phenotype was defined by low levels of MIF, IL-8, and SCF. Secondly, the inflammatory phenotype included those cases with elevated MIF and/or IL-8/CXCL8 but low SCF levels. Thirdly, SCF elevation was prioritized in the classification algorithm (phenotype 3) due to its hypothesized role in tumor and metastatic spread (dissemination phenotype). The distribution of phenotypes was 18.3% low-inflammatory, 26.7% inflammatory, and 55.0% dissemination (see Table 4).

Analysis of systemic inflammation parameters presented a significant difference among the three phenotypes, as presented in Table 4. Significant increases in the leucocyte and neutrophil count, as well as the ESR and fibrinogen serum levels, were noted in phenotype 2 (previously defined as the inflammatory one).

### 3.6. Predictors of Metastatic Disease

**Figure 5:**
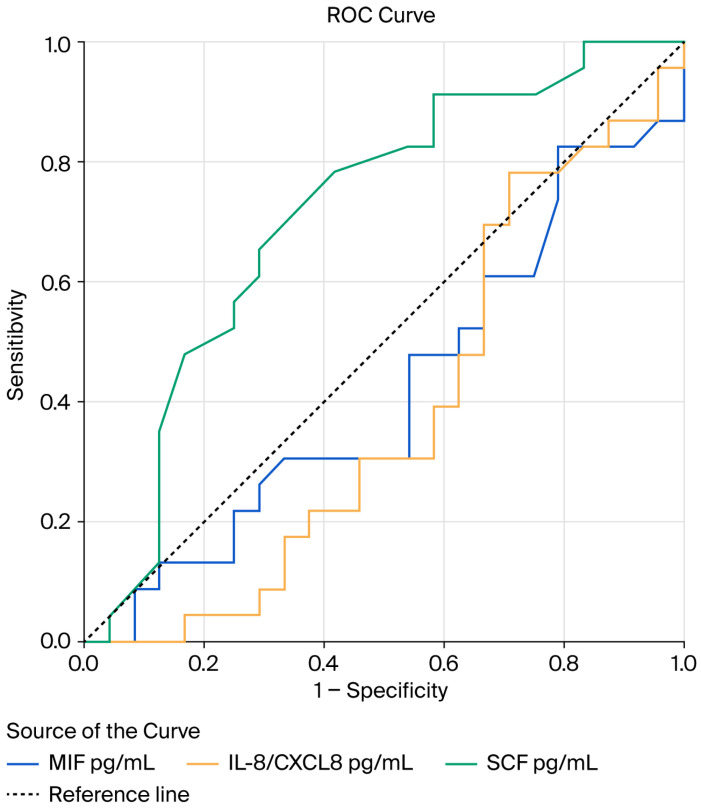
Receiver operating characteristic (ROC) curve analysis illustrating the discriminatory performance of circulating interleukin-8 (IL-8/CXCL8), macrophage migration inhibitory factor (MIF), and stem cell factor (SCF) for identification of metastatic disease. SCF demonstrated a significant discriminatory ability, with an area under the curve (AUC) of 0.712 (95% CI 0.561–0.863, *p* = 0.013). In contrast, MIF and IL-8 showed no significant predictive performance, with AUC values of 0.419 (95% CI 0.254–0.584, *p* = 0.344) and 0.383 (95% CI 0.220–0.547, *p* = 0.170), respectively. (Diagonal segments are produced by ties.) Abbreviations: MIF—Macrophage migration inhibitory factor; IL-8/CXCL8—Interleukin-8; SCF—Stem cell factor.

ROC curve analysis (Figure 5) illustrates the discriminatory performance of circulating IL-8/CXCL8, MIF, and SCF for the identification of metastatic disease.

SCF demonstrated moderate discriminatory ability, with an AUC of 0.712 (95% CI 0.561–0.863), which reached statistical significance (*p* = 0.013). In contrast, MIF and IL-8 showed no significant predictive performance, with AUC values of 0.419 (95% CI 0.254–0.584, *p* = 0.344) and 0.383 (95% CI 0.220–0.547, *p* = 0.170), respectively (see Figure 5).

Consistent with the ROC analysis, the univariate logistic regression showed that SCF was the only biomarker predictor of metastatic disease, both when analyzed as a continuous variable (OR 1.12, 95% CI 1.00–1.25, *p* = 0.046) or when dichotomized using the predefined cut-off (OR 4.72, 95% CI 1.36–16.39, *p* = 0.015), as presented in Table 5.

Importantly, the association of SCF with metastasis occurrence persisted in multivariable models adjusted for age and gender, where high SCF levels remained significantly associated with metastatic disease after adjustment (see Table 6).

Further, a significantly higher prevalence of metastatic disease was observed among patients with dissemination phenotype (phenotype 3) compared to those with the inflammatory one (phenotype 2): 65.3% vs. 16.7%, *p* = 0.005.

## 4. Discussion

Tumoral progression is driven by a complex interaction between aggressive, poorly differentiated malignant cells and host-impaired antitumoral immune surveillance, arising from continuous crosstalk with the surrounding microenvironment [18,19]. Within this framework, circulating cytokines are part of the tumor–host interactions and might define distinct biological patterns of tumoral behavior. By simultaneously evaluating MIF, IL-8/CXCL8, and SCF in patients with pancreatic cancer, we found that the concept of inflammatory activation and metastatic potential might have partially independent pathways. Accordingly, the originality of the study presented here lies in differentiating a non-specific, proportional low-grade inflammatory response from biomarker patterns associated with tumor aggressiveness potential.

This dissociation between inflammatory signaling and metastatic potential might be particularly relevant in pancreatic adenocarcinoma with aggressive biological features coexisting with profound systemic inflammation [18,19,21,43].

MIF is a pleiotropic cytokine ubiquitously expressed, involved in inflammatory, immune, and tumorigenesis processes [21,22,23,24,27]. MIF is a stress-response cytokine that can be induced by glucocorticoids, yet paradoxically counter-regulates their immunosuppressive effects [25]. In this context, given the corticosteroids’ central role in metabolic homeostasis and insulin regulation, the MIF-mediated beta-cell function may reflect not only direct cellular effects, but also broader dysregulation of stress-related endocrine pathways [25,28].

MIF, a product of activated macrophages, sustains macrophage survival and function [30]; stimulates cytokines induction, including tumor necrosis factor (TNF)-alfa, interleukin (IL)-1, and interleukin (IL)-6 [23]; has a critical role for NLRP3 inflammasome activation [30]; suppresses the p53-dependent apoptosis [28,44]; and promotes leukocytes migration and recruitment in inflammatory reactions [22]. In regard to inflammatory pathways, MIF levels were found to be increased in different non-malignant pathologies like inflammatory arthritis, glomerulonephritis, or sepsis [22,28]. In line with these established MIF inflammatory roles, our findings showed that elevated MIF levels in patients with pancreatic cancer were associated with higher leukocyte and neutrophil counts and increased fibrinogen levels.

Further, multiple studies have reported upregulated MIF expression across a wide range of malignancies, such as prostate, ovarian, breast, esophageal, hepatocellular, bladder, non-melanoma skin, colorectal, or lung cancer [21,22]. Potential pathogenic mechanisms that might explain the MIF role in cancer include receptor-mediated signaling via CD74 (often in complex with CXCR2/CXCR4), negative regulation of the p53-mediated cellular growth arrest and apoptosis [26,44], upregulation of TLR4 expression [10], ERK1/ERK2 signaling activation, inhibition of the JAB1 activity [10], and enhancement of the IL-2–dependent Treg axis [27].

Accumulating evidence indicates that MIF expression, both serum levels and tissue expression, is increased in pancreatic adenocarcinoma when compared to healthy controls and might be associated with a more aggressive tumor biology [21,45,46]. In this regard, in our study, even if we did not identify an association between serum MIF and metastatic disease, the proportion of cases with high MIF levels was associated with larger tumoral dimensions. It is also of note that in previous reports, the tumoral aggressiveness associated with MIF expression was primarily defined by unfavorable biological and clinical features rather than metastatic spread [21,45,46].

Interestingly, MIF impairs beta-cells by decreasing L-type calcium channel activity, reducing the protein expression of the channel’s alpha-1 subunit, and increasing p-Src activity. Therefore, MIF might interfere with beta-cell function, causing dysfunction of insulin secretion in beta-cells, leading to potential new-onset diabetes in pancreatic adenocarcinoma [47]. Studies on animal models showed that MIF is directly involved in the control of glucose homeostasis [48]. MIF deficiency leads to age-dependent impairment of glucose homeostasis in mice. MIF stimulates the recruitment of adipose tissue macrophages during obesity, promoting local inflammation and insulin resistance [49].

Even if a relation with new-onset diabetes was found in our dataset, the design of the study presented here cannot sustain a causal relationship with the occurrence of new-onset diabetes, given its cross-sectional nature and single-time-point biomarker assessment. Our findings should be interpreted as associative rather than indicative of a pathogenic sequence. Although speculative, these findings suggest that elevated MIF may reflect an initial phase during which pancreatic beta-cell dysfunction and injury occur; however, when diabetes becomes clinically manifest, inflammatory activity shifts toward parallel pathways independent of overt MIF elevation.

IL-8/CXCL8 was initially identified as a neutrophil chemotactic factor, and was only later recognized for its role in cancer progression [19]. Several studies have reported higher serum IL-8/CXCL8 in different cancers when compared to healthy subjects [33,35].

Elevated levels of IL-8/CXCL8 have been associated with a poorer outcome in several tumors and have been related to advanced diseases, treatment resistance, and possibly promoting neoangiogenesis and immune cell recruitment [33]. Additionally, the tumor microenvironment has been shown to contribute to tumor progression [19].

IL-8/CXCL8 acts as a key mediator of a permissive tumoral microenvironment, integrating complex interactions involved in angiogenesis, immune cell recruitment, tumor cell migration, and survival [18,31,50]. Evidence suggests that chemokines, including IL-8/CXCL8, are implicated in resistance to both chemotherapy and molecularly targeted agents [31,36]. Nevertheless, a clear association between IL-8/CXCL-8 levels and cancer survival has not been consistently demonstrated [32,51]. Further, elevated IL-8/CXCL8 levels have been reported in pancreatic cancer compared to non-malignant conditions [32,34,50].

In patients with chronic liver disease and obstructive jaundice, increased IL-8/CXCL8 has been implicated in portal inflammation and fibrosis development [52]. In addition, although hyperbilirubinemia is associated with systemic inflammatory activation in obstructive cholestasis, direct evidence linking bilirubin levels to circulating IL-8 concentrations in pancreatic cancer is limited [32]. We herein identified higher direct and total bilirubin levels in pancreatic cancer patients presenting with high MIF serum values. In a study on pancreatic ductal adenocarcinoma, IL-8/CXCL8 was identified as a stratification factor, showing better performance in the overall cohort after excluding patients with jaundice, despite the absence of a significant correlation between IL-8 and bilirubin levels [32]. Moreover, although hyperbilirubinemia might be an important confounding factor in the evaluation of inflammatory biomarkers, IL-8/CXCL8 appears to be relatively independent of bilirubin fluctuations [32].

However, the potential confounding effect of hyperbilirubinemia on circulating cytokines should be further explored in larger studies, as the secondary obstructive cholestasis might trigger the activation of inflammatory pathways.

Pancreatic cancer has one of the highest rates of cachexia, driven by the tumor–host interactions and sustained systemic inflammation. This process reflects rather active disease biology and is closely linked to inflammatory signaling [50]. Our analysis showed that patients with significant weight loss before inclusion had higher IL-8/CXCL8 serum levels: 10.6 (5.4; 37.6) versus 4.2 (1.5; 34.7) pg/mL, reaching borderline statistical significance (*p* = 0.050). As a pro-inflammatory chemokine, IL-8/CXCL8 was shown to contribute to weight loss [35], especially due to catabolic responses and muscle wasting [36] secondary to metabolic imbalance and protein degradation in the context of persistent cytokine signaling [35,36]. It was shown that IL-8/CXCL8 secreted by pancreatic cancer cells and tumor-associated stromal cells activates the CXCR2–ERK1/2 signaling pathway, leading to skeletal muscle atrophy [50].

Among the inflammatory markers analyzed in this research, none were a strong predictor of metastasis; the SCF proved to be associated with metastatic disease in pancreatic adenocarcinoma. Moreover, in contrast to the other two markers assessed, there were no significant correlations between the SCF levels and any of the inflammatory parameters tested. Therefore, SCF emerged as a distinct parameter, remaining significant in both univariable and multivariable regression models, associated with tumor invasiveness when defined by metastasis occurrence.

The SCF/c-KIT system is overactivated during oncogenic mutagenesis due to gain-of function mutations or receptor overexpression [39,40] and is associated not only with the onset, but also with the progression of several cancers [37,39,42,53]. Also, higher SCF-expression was associated with lower survival rates [37].

It was hypothesized that the SCF/c-KIT system might have a growth-regulating role in the normal pancreas, which, when dysfunctional, leads to upregulation and malignant transformation [41]. Furthermore, SCF is involved in pancreatic cancer cells’ differentiation into insulin-producing cells [37].

Taken together, our findings suggest the hypothesis that the inflammatory and metastatic signaling pathway might have partially independent evolution in pancreatic cancer. Therefore, while an inflammatory pathway might be characterized by MIF and IL-8/CXCL8, SCF might identify the metastasis dissemination-dominant profile. However, it is also important to note that we did not identify a relation of MIF or IL8/CXCL8 with metastasis, and that the SCF levels were not correlated with inflammation.

The research presented here has some limitations. First, it was conducted in a single-center setting with a relatively small sample size, which may limit the generalization of the findings and raise concerns regarding statistical stability and external validity. In this context, the multivariate analysis was also underpowered, adjusted only for age and gender, and therefore, the risk of overfitting or residual confounding cannot be excluded. Hence, a formal a priori sample size calculation was not performed. The cross-sectional design with measurement at a single time-point of the biomarkers does not allow the evaluation of temporal changes and causal relations. In addition, although methodologically convenient in exploration settings, the use of median-derived cut-offs for the biomarkers tested might lead to biased results. Another limitation of the study is the incomplete characterization of all metastatic sites. Further, the results presented here are only hypothesis-generating and require validation in larger, multicenter studies.

Although population-based studies report a modest increase in pancreatic cancer incidence in males, the slight female predominance observed in our cohort likely reflects the sample variability. Therefore, gender was included as a covariate in multivariate analyses to account for any potential confounding effect. Despite these limitations, the present study has several notable strengths. The study had a prospective design with consecutive patients’ enrollment, reducing the selection bias and enhancing internal validity. The biomarkers were measured at the same time, with all samples in a single analytical run, minimizing the inter-assay variability. Importantly, this study adds new insights by analyzing three biomarkers for which limited data are currently available. Furthermore, the identification of distinct inflammatory and dissemination phenotypes represents a hypothesis-generating step with potential relevance in pancreatic cancer risk stratification.

The originality of this research resides not only in evaluating less-explored biomarkers in pancreatic cancer, but also in introducing a conceptual framework where the kinetics of different tumor-related biomarkers may delineate distinct biological and clinical patterns of disease progression, without implying causality. The panel of biomarkers chosen for our study reveals the dynamic of the complex biology and physiopathology of pancreatic cancer. IL-8, already a classical pro-inflammatory biomarker, was shown to be a promising tool in diagnosing pancreatic cancer in combination with CA19-9, and CEACAM6 [32], while SCF promotes stem cells recruitment, proliferation and invasion of pancreatic cancer cells [42,54]. On the other hand, MIF was identified by previous research as a key promoter of metastasis and disease aggressiveness by inhibiting the orphan nuclear receptor NR3C2 [55] and promoting epithelial to mesenchymal transition [45]. Thus, by analyzing these three biomarkers simultaneously, our research can offer new perspectives toward the identification of disease phenotypes. Their measurement and evolution can provide, over time, a more informative picture of the underlying mechanisms of pancreatic cancer.

## 5. Conclusions

In conclusion, our results support the notion that pancreatic cancer heterogeneity can be partially explained by distinct cytokine-driven phenotypes. MIF and IL-8/CXCL8 were primarily related to inflammatory pathways, while SCF emerged as a marker for metastatic disease. The novelty of this study lies in the idea of describing disease phenotypes that may enable new therapeutic directions.

Elevated MIF and IL-8/CXCL8 levels were additionally associated with diabetes mellitus prevalence, potentially reflecting cytokine-mediated impairment of metabolic homeostasis in pancreatic cancer. Further, IL-8/CXCL8 was associated with cholestasis and cancer-related weight loss, consistent with its known inflammation-driven and cancer-associated cachexia.

Altogether, these findings add to the growing evidence that cytokine signaling contributes to clinically relevant heterogeneity in pancreatic cancer.

## Figures and Tables

**Table 1:** Baseline characteristics of patients included.

Parameter	Cases n = 60
Gender, M/F (%M)	26/34 (43.3)
Age, years med (q1; q3)	69.5 (64.2; 76.0)
BMI, kg/m^2^ med (q1; q3)	24.7 (21.9; 28.0)
Smoke, n (%)	16/41 (39.0)
Alcohol, n (%)	15/42 (26.3)
Meat-rich diet, n (%)	20/36 (35.7)
Sweet-rich diet, n (%)	16/41 (28.1)
High-fat diet, n (%)	13/44 (22.8)
Abdominal pain, n (%)	40/14 (74.1)
Nausea, n (%)	22/30 (42.3)
Diarrhea, n (%)	13/42 (23.6)
Weight loss, n (%)	41/13 (75.9)
Weight loss, kg med (q1; q3)	10.0 (4.0; 15.0)
Diabetes mellitus, n (%)	24/57 (42.1)
MIF, pg/mL med (q1; q3)	4.4 (2.6; 9.0)
MIF, cut off pg/mL n (%)	29/31 (48.3)
IL-8/CXCL8, pg/mL med (q1; q3)	7.5 (3.8; 35.5)
IL-8/CXCL8, cut-off pg/mL n (%)	30/30 (50.0)
SCF, pg/mL med (q1; q3)	13.1 (9.5; 17.4)
SCF, cut-off pg/mL n (%)	33/27 (55.0)
Leucocytes/μL, med (q1; q3)	8.1 (6.6; 10.9)
Neutrophils/μL, med (q1; q3)	5.5 (4.4; 8.0)
Lymphocytes/μL, med (q1; q3)	1.6 (1.1; 2.1)
Hemoglobin, g/dL med (q1; q3)	12.4 (11.3; 13.7)
Thrombocytes/μL, med (q1; q3)	280.0 (205.5; 344.5)
ESR, mm/h med (q1; q3)	31.0 (20.7; 63.7)
CRP, mg/L med (q1; q3)	14.5 (4.9; 86.0)
Fibrinogen, mg/dL med (q1; q3)	645.0 (539.0; 801.0)
Total bilirubin, mg/dL med (q1; q3)	1.4 (0.5; 7.4)
Direct bilirubin, mg/dL med (q1; q3)	0.7 (0.1; 5.0)
Creatinine, mg/dL med (q1; q3)	0.7 (0.6; 0.9)
Uric acid, mg/dL med (q1; q3)	4.4 (3.7; 5.7)
ASAT, U/L med (q1; q3)	54.0 (26.0; 140.0)
ALAT, U/L med (q1; q3)	56.5 (20.0; 186.5)
GGT, U/L med (q1; q3)	308.5 (38.5; 996.5)
ALP, U/L med (q1; q3)	197.0 (82.5; 492.5)
Glycemia, mg/dL med (q1; q3)	120.0 (104.0; 144.0)
Amylase, U/L med (q1; q3)	60.0 (36.0; 93.5)
Lipase U/L med (q1; q3)	40.5 (18.5; 105.5)
Total cholesterol, mg/dL, med (q1; q3)	191.0 (122.7; 214)
Triglycerides, mg/dL med (q1; q3)	112.0 (94.0; 223.0)
Large tumor diameter, n (%)	25/22 (53.2)
Large tumor dimension, n (%)	14/23 (37.8)
Local tissue invasion, n (%)	40/6 (87.0)
Local vascular invasion, n (%)	29/15 (65.9)
Metastatic disease, n (%)	23/24 (48.9)
Fatigue, med (q1; q3)	34.0 (14.7; 40.7)

Abbreviations: ALAT—alanine aminotransferase; ALP—alkaline phosphatase; ASAT—aspartate aminotransferase; CRP—C-reactive protein; ESR—erythrocyte sedimentation rate; HDL—high-density lipoprotein; GGT—gamma-glutamyl transferase; IL-8/CXCL8—Interleukin-8; MIF—macrophage migration inhibitory factor; sCF—Stem cell factor.

**Table 2:** (**a**) Clinical and laboratory parameters showing statistically significant differences according to macrophage migration inhibitory factor (MIF) serum level. (**b**) Clinical and laboratory parameters showing statistically significant differences according to interleukin-8 (IL-8/CXCL8) serum levels. (**c**) Clinical and laboratory parameters showing statistically significant differences according to stem cell factor (SCF) serum levels.

**(a)**				
Parameter	Cases n = 60	Low MIF (≤5.2 pg/mL) n = 31	High MIF (>5.2 pg/mL) n = 29	*p*-value *
New-onset diabetes, n (%)	10/47 (17.5)	8/21 (27.5)	2/26 (7.1)	0.042
MIF, pg/mL med (q1; q3)	4.4 (2.6; 9.0)	2.6 (2.1; 3.5)	9.3 (7.0; 17.3)	0.000
IL-8/CXCL8, pg/mL med (q1; q3)	7.5 (3.8; 35.5)	4.5 (2.7; 7.4)	32.0 (8.8; 47.6)	0.000
IL-8/CXCL8 cut-off 10.6 pg/mL n (%)	30/30 (50.0)	5/26 (16.1)	21/8 (72.4)	0.000
Leucocytes/μL, med (q1; q3)	8.1 (6.6; 10.9)	7.2 (5.7; 8.5)	10.2 (8.1; 11.7)	0.003
Neutrophils/μL, med (q1; q3)	5.5 (4.4; 8.0)	4.9 (3.9; 5.7)	6.8 (5.2; 8.6)	0.005
Fibrinogen mg/dL med (q1; q3)	645.0 (539.0; 801.0)	599.0 (504.0; 744.0)	689.0 (597.2; 860.2)	0.038
Larger tumor dimension, n (%)	14/23 (37.8)	4/15 (21.1)	10/8 (34.5)	0.030
**(b)**				
Parameter	Cases n = 60	Low IL-8/CXCL8 (≤10.6 pg/mL) n = 34	High IL-8/CXCL8 (>10.6 pg/mL) n = 26	*p*-value *
Diarrhea, n (%)	13/42 (23.6)	4/30 (11.8)	9/25 (36.0)	0.049
Long-standing diabetes, n (%)	14/43 (24.5)	4/27 (12.9)	10/16 (38.5)	0.026
New-onset diabetes, n (%)	10/47 (17.5)	9/22 (29.0)	1/25 (3.8)	0.013
MIF, pg/mL med (q1; q3)	4.4 (2.6; 9.0)	3.1 (2.1; 4.8)	9.5 (5.8; 16.9)	0.000
MIF, cut off 5.2 pg/mL n (%)	29/31 (48.3)	8/26 (23.5)	21/5 (80.8)	0.000
IL-8/CXCL8, pg/mL med (q1; q3)	7.5 (3.8; 35.5)	4.2 (2.0; 6.1)	37.6 (18.8; 56.7)	0.000
ESR mm/h, med (q1; q3)	31.0 (20.7; 63.7)	29.0 (19.0; 50.0)	67.0 (25.5; 113.0)	0.034
CRP mg/L, med (q1; q3)	14.5 (4.9; 86.0)	9.5 (2.5; 27.2)	55.7 (5.5; 142.0)	0.035
Fibrinogen mg/dL med (q1; q3)	645.0 (539.0; 801.0)	588.0 (509.5; 770.5)	709.5 (618.5; 872.5)	0.019
Total bilirubin, mg/dL med (q1; q3)	1.4 (0.5; 7.4)	0.6 (0.4; 4.8)	4.0 (0.8; 12.9)	0.005
Direct bilirubin, mg/dL med (q1; q3)	0.7 (0.1; 5.0)	0.15 (0.1; 2.7)	2.4 (0.2; 7.3)	0.009
**(c)**				
Parameter	Cases n = 60	Low SCF (≤13.1 pg/mL) n = 27	High SCF (>13.1 pg/mL) n = 33	*p*-value *
SCF, pg/mL med (q1; q3)	13.1 (9.5; 17.4)	9.5 (7.7; 11.9)	16.5 (14.2; 22.6)	0.000
Creatinine, mg/dL med (q1; q3)	0.7 (0.6; 0.9)	0.6 (0.5; 0.7)	0.8 (0.7; 1.0)	0.010
Metastatic disease, n (%)	23/24 (48.9)	6/15 (28.6)	17/9 (65.4)	0.012

* *p*-values were derived from the Mann–Whitney U test for continuous variables and the chi-square test for dichotomous ones. *p*-value ≤ 0.05 was considered statistically significant. Abbreviations: CRP—C-reactive protein; ESR—erythrocyte sedimentation rate; IL-8/CXCL8—interleukin-8; MIF—macrophage migration inhibitory factor; SCF—stem cell factor.

**Table 3:** Correlations between the circulating markers (macrophage migration inhibitory factor, interleukin-8, and stem cell factor) and systemic inflammatory markers.

	IL-8/CXCL8 (pg/mL)	SCF (pg/mL)	ESR (mm/h)	CRP (mg/L)	Fibrinogen (mg/dL)	Leucocytes (/μL)	Neutrophils (/μL)	Lymphocytes (/μL)
MIF (pg/mL)	* *p* = 0.000	* *p* = 0.051	* *p* = 0.109	* *p* = 0.015	* *p* = 0.037	* *p* = 0.009	* *p* = 0.018	* *p* = 0.614
	rho = 0.583	rho = 0.697	rho = 0.336	rho = 0.356	rho = 0.336	rho = 0.345	rho = 0.311	rho = 0.069
IL-8/CXCL8 (pg/mL)	-	* *p* = 0.995	* *p* = 0.055	* *p* = 0.052	* *p* = 0.011	* *p* = 0.266	* *p* = 0.133	* *p* = 0.443
		rho = 0.001	rho = 0.397	rho = 0.288	rho = 0.403	rho = 0.150	rho = 0.201	rho = −0.105
SCF (pg/mL)	-	-	* *p* = 0.007	* *p* = 0.211	* *p* = 0.236	* *p* = 0.636	* *p* = 0.194	* *p* = 0.404
			rho = −0.536	rho = −0.188	rho = −0.194	rho = 0.064	rho = 0.175	rho = 0.114

* *p*-values were derived from the bivariate rho’s Spearman correlation analysis; *p*-value ≤ 0.05 was considered statistically significant. Abbreviations: MIF—Macrophage migration inhibitory factor; IL-8/CXCL8—Interleukin-8; SCF—Stem cell factor; ESR—Erythrocyte sedimentation rate; CRP—C-reactive protein; rho—Spearman’s rank correlation coefficient.

**Table 4:** Comparison of inflammatory parameters across the three phenotypes defined by the cytokine kinetics.

	Phenotype 1 (Low-Inflammatory)	Phenotype 2 (Inflammatory)	Phenotype 3 (Dissemination)	
	MIF Low and IL-8/CXCL8 Low and SCF Low	MIF High and/or IL-8/CXCL8 High and SCF Low	MIF Low or High and IL-8/CXCL8 Low or High and SCF High	
	11/49 (18.3)	16/44 (26.7)	33/27 (55.0)	*p*-Value *
ESR mm/h, med (q1; q3)	31.5 (8.5; 48.5)	111.0 (86.0; 117.5)	26.0 (20.0; 32.0)	0.004
CRP mg/L, med (q1; q3)	10.2 (6.8; 16.6)	74.0 (11.1; 148.8)	11.2 (3.0; 68.4)	0.094
Fibrinogen mg/dL, med (q1; q3)	547.5 (464.2; 850.2)	852.0 (588.0; 904.0)	623.0 (537.7; 718.7)	0.043
Leucocytes/μL, med (q1; q3)	7.0 (5.4; 7.7)	10.4 (6.6; 13.6)	8.5 (6.8; 10.8)	0.018
Neutrophils/μL, med (q1; q3)	4.4 (3.8; 5.3)	7.0 (4.5; 9.4)	5.6 (4.9; 8.1)	0.010

* *p*-values were derived from the Kruskal–Wallis test. *p*-value < 0.05 was considered statistically significant. Abbreviations: CRP—C-reactive protein; ESR—erythrocyte sedimentation rate; IL-8/CXCL8—interleukin-8; MIF—macrophage migration inhibitory factor; SCF—stem cell factor.

**Table 5:** Univariate logistic regression analysis of biomarkers for metastatic disease.

Predictor	OR	95% CI	*p*-Value *
MIF, pg/mL	0.96	0.88–1.05	0.398
high MIF, cut-off 5.2 pg/mL	0.54	0.17–1.74	0.304
IL-8/CXCL8, pg/mL	0.97	0.94–1.00	0.074
high IL-8/CXCL8, cut-off 10.6 pg/mL	0.46	0.14–1.48	0.194
SCF, pg/mL	1.12	1.00–1.25	0.046
high SCF, cut-off 13.1 pg/mL	4.72	1.36–16.39	0.015

* *p*-value ≤ 0.05 was considered statistically significant. Abbreviations: IL-8/CXCL8—Interleukin-8; MIF—Macrophage migration inhibitory factor; SCF—Stem cell factor.

**Table 6:** Multivariable logistic regression analysis for metastatic disease.

Predictor	ORs	95% CI	*p*-Value *
SCF, pg/mL	1.12	1.00–1.26	0.052
high SCF, cut-off 13.1 pg/mL	4.81	1.34–17.25	0.016
IL-8/CXCL8, pg/mL	1.02	0.99–1.05	0.193

* Adjusted odds ratios (ORs) for age and gender with 95% confidence intervals (CI) were obtained from multivariable logistic regression models including age and sex as covariates; *p*-value ≤ 0.05 was considered statistically significant. Abbreviations: IL-8/CXCL8—Interleukin-8; SCF—Stem cell factor.

## Data Availability

Data is contained within the article or Supplementary Materials. Further inquiries can be directed to the corresponding author.

## Supplementary Materials

The following supporting information can be downloaded at https://www.mdpi.com/article/10.3390/diagnostics16091373/s1. Table S1: Baseline characteristics of the patients with pancreatic cancer, according to the macrophage migration inhibitory factor (MIF) serum levels; Table S2. Baseline characteristics of the patients with pancreatic cancer, according to the interleukine-8 (IL-8/CXCL8) serum levels; Table S3. Baseline characteristics of the patients with pancreatic cancer, according to the stem cell factor (SCF) serum levels; Table S4. Pancreatic cancer patients’ characteristics stratified by the metastatic status.

## Abbreviations

The following abbreviations are used in this manuscript:

ALAT	Alanine aminotransferase
ALP	Alkaline phosphatase
ASAT	Aspartate aminotransferase
CRP	C-reactive protein
ESR	Erythrocyte sedimentation rate
HDL	High-density lipoprotein
GGT	Gamma-glutamyl transferase
IL-8/CXCL8	Interleukin-8/(CXC)-Motiv-Chemokine ligand 8
MIF	Macrophage migration inhibitory factor
SCF	Stem cell factor
