# Supplementary material for: Distinct Inflammatory and Dissemination Signatures Defined by Macrophage Migration Inhibitory Factor (MIF), Interleukin-8 (IL-8/CXCL8), and Stem Cell Factor (SCF) in Pancreatic Adenocarcinoma
Source: Diagnostics (Basel). 2026 Apr 30;16(9):1373. doi: 10.3390/diagnostics16091373 (PMC13164223; doi:10.3390/diagnostics16091373)
Supplement: Supplementary file 1 [file diagnostics-16-01373-s001.zip › diagnostics-4125873-supplementary.pdf]

Table S1. Baseline characteristics of the patients with pancreatic cancer, according to the macrophage migration inhibitory factor (MIF) serum levels

**Parameter**	**Cases** n=60	**Low MIF** (<=5.2 pg/mL) n=31	**High MIF** (>5.2 pg/mL) n=29	**p-value***
Gender, M/F (%M)	26/ 34 (43.3)	10/ 21 (32.3)	16/ 13 (55.2)	0.073
Age, years med (q1; q3)	69.5 (64.2; 76.0)	69.0 (56.0; 77.0)	70.0 (66.5; 76.0)	0.599
BMI, kg/m^2 med (q1; q3)	24.7 (21.9; 28.0)	24.9 (22.7; 28.7)	24.0 (21.8; 28.0)	0.604
Smoke, n (%)	16/ 41 (39.0)	8/ 21 (27.6)	8/ 20 (28.6)	0.381
Alcohol, n (%)	15/ 42 (26.3)	5/ 24 (17.2)	10/ 18 (35.7)	0.113
Meat-rich diet, n (%)	20/ 36 (35.7)	11/ 1(39.3)	9/ 19 (32.1)	0.577
Sweet-rich diet, n (%)	16/ 41 (28.1)	5/ 24 (17.2)	9/ 19 (31.0)	0.064
High-fat diet, n (%)	13/ 44 (22.8)	6/ 23 (20.7)	11/ 17 (37.9)	0.698
Abdominal pain, n (%)	40/ 14 (74.1)	18/ 9 (66.7)	7/ 21 (24.1)	0.214
Nausea, n (%)	22/ 30 (42.3)	11/ 16 (40.7)	11/ 14 (44.0)	0.812
Diarrhea, n (%)	13/ 42 (23.6)	6/ 21 (22.2)	7/ 21 (25.0)	0.808
Weight loss, n (%)	41/ 13 (75.9)	18/8 (69.2)	23/5 (79.3)	0.267
Weight loss, kg med (q1; q3)	10.0 (4.0; 15.0)	8.0 (1.0; 10.0)	10.0 (5.0; 15.0)	0.199
Diabetes mellitus, n (%)	24/ 33 (42.1)	12/ 17 (41.4)	12/ 16 (42.9)	0.910
Long-standing diabetes, n (%)	14/ 43 (23.3)	4/ 25 (13.8)	10/ 18 (35.7)	0.055
New-onset diabetes, n (%)	10/ 47 (16.6)	8/ 21 (27.6)	2/ 26 (7.1)	0.042
MIF, pg/mL med (q1; q3)	4.4 (2.6; 9.0)	2.6 (2.1; 3.5)	9.3 (7.0; 17.3)	0.000
MIF, cut off	29/ 31 (48.3)	-	-	-
IL-8, pg/mL med (q1; q3)	7.5 (3.8; 35.5)	4.5 (2.7; 7.4)	32.0 (8.8; 47.6)	0.000
IL-8 cut-off pg/mL	30/ 30 (50.0)	5/ 26 (16.1)	21/ 8 (72.4)	0.000
SCF, pg/mL med (q1; q3)	13.1 (9.5; 17.4)	13.1 (10.7; 18.8)	13.1 (8.3; 16.5)	0.428
SCF, cut-off pg/mL	33/ 27 (55.0)	17/ 14 (54.8)	16/ 13 (55.2)	0.979
Leucocytes/µL, med (q1; q3)	8.1 (6.6; 10.9)	7.2 (5.7; 8.5)	10.2 (8.1; 11.7)	0.003
Neutrophils/µL, med (q1; q3)	5.5 (4.4; 8.0)	4.9 (3.9; 5.7)	6.8 (5.2; 8.6)	0.005
Lymphocytes/µL, med (q1; q3)	1.6 (1.1; 2.1)	1.4 (1.2; 1.9)	1.6 (0.9; 2.2)	0.767
Hemoglobin g/dL, med (q1; q3)	12.4 (11.3; 13.7)	12.3 (11.3; 13.4)	12.6 (11.8; 14.5)	0.209
Thrombocytes/µL, med (q1; q3)	280.0 (205.5; 344.5)	270.5 (199.0; 323)	301.0 (216.0; 355.0)	0.260
ESR mm/h, med (q1; q3)	31.0 (20.7; 63.7)	32.0 (19.0; 57.0)	30.0 (25.5; 86.5)	0.245
CRP mg/L, med (q1; q3)	14.5 (4.9; 86.0)	9.9 (2.6; 33.8)	66.8 (5.8; 126.7)	0.059
Fibrinogen mg/dL med (q1; q3)	645.0 (539.0; 801.0)	599.0 (504.0; 744.0)	689.0 (597.2; 860.2)	0.038
Total bilirubin, mg/dL med (q1; q3)	1.4 (0.5; 7.4)	0.9 (0.5; 5.9)	3.6 (0.6; 11.8)	0.140
Direct bilirubin, mg/dL med (q1; q3)	0.7 (0.1; 5.0)	0.3 (0.1; 3.8)	2.1 (0.1; 7.6)	0.091
Creatinine, mg/dL med (q1; q3)	0.7 (0.6; 0.9)	0.7 (0.5; 0.8)	0.8 (0.6; 1.0)	0.186
Uric acid, mg/dL med (q1; q3)	4.4 (3.7; 5.7)	4.0 (3.5; 5.4)	4.8 (3.9; 5.8)	0.214
ASAT U/L, med (q1; q3)	54.0 (26.0; 140.0)	32.0 (23.0; 136.0)	93.0 (33.0; 147.0)	0.119
ALAT U/L, med (q1; q3)	56.5 (20.0; 186.5)	49.5 (20.0; 272.5)	64.0 (35.7; 186.5)	0.494

GGT U/L, med (q1; q3)	308.5 (38.5; 996.5)	297.0 (34.2; 1019.7)	308.5 (57.7; 854.7)	0.714
ALP U/L, med(q1; q3)	197.0 (82.5; 492.5)	159.5 (73.2; 347.2)	398.0 (103.0; 616.5)	0.125
Glycemia, mg/dL med (q1; q3)	120.0 (104.0; 144.0)	120.0 (103.5; 142.0)	124.0 (104.5; 147.5)	0.544
Amylase U/L, med (q1; q3)	60.0 (36.0; 93.5)	66.0 (50.7; 111.2)	38.0 (29.0; 70.0)	0.008
Lipase U/L, med (q1; q3)	40.5 (18.5; 105.5)	40.5 (21.5; 207.7)	39.5 (13.0; 98.5)	0.662
Total cholesterol mg/dL, med (q1; q3)	191.0 (122.7; 214)	200.0 (131.0; 241.0)	157.0 (92.0; 199.5)	0.210
Triglycerides mg/dL, med (q1; q3)	112.0 (94.0; 223.0)	112.0 (95.0; 214.5)	110.0 (76.7; 287.5)	0.940
Large tumor diameter, n (%)	25/ 22 (53.2)	12/ 15 (44.4)	11/ 9 (37.9)	0.147
Large tumor dimension, n (%)	14/ 23 (37.8)	4/ 15 (21.1)	10/ 8 (34.5)	0.030
Local tissular invasion, n (%)	40/ 6 (87.0)	21/ 3 (87.5)	19/ 3 (86.4)	0.909
Local vascular invasion, n (%)	29/ 15 (65.9)	15/ 9 (62.5)	14/ 6 (70.0)	0.601
Metastatic disease, n (%)	23/ 24 (48.9)	14/ 11 (56.0)	9/ 13 (31.0)	0.302
Fatigue, med (q1; q3)	34.0 (14.7; 40.7)	27.5 (14.0; 37.7)	35.5 (24.0; 43.7)	0.112

***** p-values were derived from the Mann–Whitney U test for continuous variables and the chi-square test for dichotomous ones. p-value < 0.05 was considered statistically significant.

Abbreviations: ALAT - alanine aminotransferase; ALP - alkaline phosphatase; ASAT - aspartate aminotransferase; CRP - C-reactive protein; ESR - erythrocyte sedimentation rate; HDL - high-density lipoprotein; GGT - gamma-glutamyl transferase; IL-8 - Interleukine-8; MIF - Macrophage migration inhibitory factor; SCF - Stem cell factor;

Table S2. Baseline characteristics of the patients with pancreatic cancer, according to the interleukine-8 (IL-8/ CXCL8) serum levels

**Parameter**	**Cases**<br><br>**n=60**	**Low IL-8/ CXCL8**<br><br>(<= 10.6 pg/mL)<br>n=34	**High IL-8/ CXCL8**<br><br>(> 10.6 pg/mL)<br>n=26	**p-value***
Gender, M/F (%M)	26/ 34 (43.3)	13/ 21 (38.2)	13/ 13 (50.0)	0.362
Age, years med (q1; q3)	69.5 (64.2; 76.0)	68.5 (56.0; 75.2)	68.5 (56.0; 75.2)	0.244
BMI, kg/m^2 med (q1; q3)	24.7 (21.9; 28.0)	23.9 (21.3; 27.5)	23.9 (21.3; 27.5)	0.321
Smoke, n (%)	16/ 41 (39.0)	11/ 22 (33.3)	5/ 19 (20.8)	0.414
Alcohol, n (%)	15/ 42 (26.3)	8/ 25 (24.2)	7/ 17 (29.2)	0.677
Meat-rich diet, n (%)	20/ 36 (35.7)	13/ 19 (40.6)	7/ 17 (29.2)	0.376
Sweet-rich diet, n (%)	16/ 41 (28.1)	8/ 25 (24.2)	8/ 16 (33.3)	0.451
High-fat diet, n (%)	13/ 44 (22.8)	7/ 26 (21.2)	6/ 18 (25.0)	0.736
Abdominal pain, n (%)	40/ 14 (74.1)	21/ 9 (70.0)	19/ 5 (79.2)	0.445
Nausea, n (%)	22/ 30 (42.3)	13/ 17 (43.3)	9/ 13 (40.9)	0.861
Diarrhea, n (%)	13/ 42 (23.6)	4/ 26 (13.3)	9/ 16 (36.0)	0.049
Weight loss, n (%)	41/ 13 (75.9)	20/ 9 (69.0)	21/ 4 (84.0)	0.198
Weight loss, kg med (q1; q3)	10.0 (4.0; 15.0)	10.0 (10.0; 0.0)	10.0 (5.7; 15.0)	0.168
Diabetes mellitus, n (%)	24/ 33 (42.1)	13/ 18 (41.9)	11/ 15 (44.0)	0.977
Long-standing diabetes, n (%)	14/ 43 (23.3)	4/ 27 (12.9)	10/ 16 (38.5)	0.026
New-onset diabetes, n (%)	10/ 47 (16.6)	9/ 22 (29.0)	1/ 25 (3.8)	0.013
MIF, pg/mL med (q1; q3)	4.4 (2.6; 9.0)	3.1 (2.1; 4.8)	9.5 (5.8; 16.9)	0.000
MIF, cut off	29/ 31 (48.3)	8/ 26 (23.5)	21/ 5 (80.8)	0.000
IL-8/ CXCL8, pg/mL med (q1; q3)	7.5 (3.8; 35.5)	4.2 (2.0; 6.1)	37.6 (18.8; 56.7)	0.000
IL-8/ CXCL8 cut-off pg/mL	30/ 30 (50.0)	-	-	0.547
SCF, pg/mL med (q1; q3)	13.1 (9.5; 17.4)	13.1 (9.5; 19.1)	13.4 (9.4; 16.5)	0.709
SCF, cut-off pg/mL	33/ 27 (55.0)	18/ 16 (52.9)	15/ 11 (57.7)	0.714
Leucocytes/µL, med (q1; q3)	8.1 (6.6; 10.9)	7.7 (6.5; 9.8)	9.5 (6.6; 12.5)	0.190
Neutrophils/µL, med (q1; q3)	5.5 (4.4; 8.0)	5.4 (4.4; 6.8)	6.5 (4.4; 9.0)	0.148
Lymphocytes/µL, med (q1; q3)	1.6 (1.1; 2.1)	1.6 (1.1; 2.0)	1.5 (0.9; 2.1)	0.615
Hemoglobin g/dL, med (q1; q3)	12.4 (11.3; 13.7)	12.4 (11.3; 13.8)	12.4 (11.7; 13.7)	0.847
Thrombocytes/µL, med (q1; q3)	280.0 (205.5; 344.5)	264.0 (195.0; 338.0)	294.0 (227.0; 380.0)	0.271
ESR mm/h, med (q1; q3)	31.0 (20.7; 63.7)	29.0 (19.0; 50.0)	67.0 (25.5; 113.0)	0.034
CRP mg/L, med (q1; q3)	14.5 (4.9; 86.0)	9.5 (2.5; 27.2)	55.7 (5.5; 142.0)	0.035
Fibrinogen mg/dL med (q1; q3)	645.0 (539.0; 801.0)	588.0 (509.5; 770.5)	709.5 (618.5; 872.5)	0.019
Total bilirubin, mg/dL med (q1; q3)	1.4 (0.5; 7.4)	0.6 (0.4; 4.8)	4.0 (0.8; 12.9)	0.005
Direct bilirubin, mg/dL med (q1; q3)	0.7 (0.1; 5.0)	0.15 (0.1; 2.7)	2.4 (0.2; 7.3)	0.009
Creatinine, mg/dL med (q1; q3)	0.7 (0.6; 0.9)	0.74 (0.6; 0.9)	0.7(0.5; 1.0)	0.811
Uric acid, mg/dL med (q1; q3)	4.4 (3.7; 5.7)	4.0 (3.5; 5.5)	4.9 (4.0; 5.7)	0.159
ASAT U/L, med (q1; q3)	54.0 (26.0; 140.0)	32.0 (22.2; 141.5)	68.5 (34.5; 141.7)	0.090

ALAT U/L, med (q1; q3)	56.5 (20.0; 186.5)	49.0 (18.5; 235.0)	64.0 (38.5; 194.0)	0.246
GGT U/L, med (q1; q3)	308.5 (38.5; 996.5)	250.0 (33.5; 1033.0)	344.0 (84.0; 761.0)	0.569
ALP U/L, med (q1; q3)	197.0 (82.5; 492.5)	124.0 (70.0; 365.0)	350.0 (120.2; 586.2)	0.134
Glycemia, mg/dL med (q1; q3)	120.0 (104.0; 144.0)	120.0 (104.0; 122.0)	128.0 (104.0; 144.0)	0.148
Amylase U/L, med (q1; q3)	60.0 (36.0; 93.5)	64.5 (48.5; 91.5)	38.0 (36.5; 104.5)	0.368
Lipase U/L, med (q1; q3)	40.5 (18.5; 105.5)	50.5 (23.7; 105.5)	30.5 (8.7; 154.0)	0.726
Total cholesterol mg/dL, med (q1; q3)	191.0 (122.7; 214)	192.0 (120.0; 216.0)	190.0 (120.0; 263.5)	0.712
Triglycerides mg/dL, med (q1; q3)	112.0 (94.0; 223.0)	107.0 (95.5; 150.5)	135.0 (76.5; 332.0)	0.662
Large tumor diameter, n (%)	25/ 22 (53.2)	15/ 13 (53.6)	10/ 9 (52.6)	0.949
Large tumor dimension, n (%)	14/ 23 (37.8)	5/ 13 (27.8)	10/ 9 (52.6)	0.219
Local tisular invasion, n (%)	40/ 6 (87.0)	20/ 3 (87.0)	20/ 3 (87.0)	1.000
Local vascular invasion, n (%)	29/ 15 (65.9)	13/ 9 (59.1)	16/ 6 (72.7)	0.340
Metastatic disease, n (%)	23/ 24 (48.9)	16/ 10 (61.5)	7/ 14 (33.3)	0.054
Fatigue, med (q1; q3)	34.0 (14.7; 40.7)	26.0 (14.0; 38.0)	36.0 (17.0; 44.0)	0.111

***** p-values were derived from the Mann–Whitney U test for continuous variables and the chi-square test for dichotomous ones. p-value < 0.05 was considered statistically significant.

Abbreviations: ALAT - alanine aminotransferase; ALP - alkaline phosphatase; ASAT - aspartate aminotransferase; CRP - C-reactive protein; ESR - erythrocyte sedimentation rate; HDL - high-density lipoprotein; GGT - gamma-glutamyl transferase; IL-8 - Interleukine-8; MIF - Macrophage migration inhibitory factor; SCF - Stem cell factor.

Table S3. Baseline characteristics of the patients with pancreatic cancer, according to the stem cell factor (SCF) serum levels

**Parameter**	**Cases**<br>**n=60**	**Low SCF**<br><br>(<=13.1 pg/mL)<br>n=27	**High SCF**<br><br>(>13.1 pg/mL)<br>n=33	**p-value***
Gender, M/F (%M)	26/ 34 (43.3)	12/ 15 (44.4)	14/ 19 (42.4)	0.875
Age, years med (q1; q3)	69.5 (64.2; 76.0)	69.0 (66.0; 73.0)	70.0 (59.5; 77.0)	0.409
BMI, kg/m^2 med (q1; q3)	24.7 (21.9; 28.0)	26.9 (23.0; 28.2)	23.3 (21.5; 26.1)	0.088
Smoke, n (%)	16/ 41 (39.0)	9/ 17 (34.6)	7/ 14 (50.0)	0.338
Alcohol, n (%)	15/ 42 (26.3)	7/ 19 (26.9)	8/ 23 (25.8)	0.924
Meat-rich diet, n (%)	20/ 36 (35.7)	9/ 17 (34.6)	11/ 19 (36.7)	0.873
Sweet-rich diet, n (%)	16/ 41 (28.1)	6/ 20 (23.1)	10/ 21 (32.3)	0.442
High-fat diet, n (%)	13/ 44 (22.8)	7/ 19 (26.9)	6/ 25 (19.4)	0.498
Abdominal pain, n (%)	40/ 14 (74.1)	18/ 7 (72.0)	22/ 7 (75.9)	0.747
Nausea, n (%)	22/ 30 (42.3)	9/ 16 (36.0)	12/ 15 (44.4)	0.746
Diarrhea, n (%)	13/ 42 (23.6)	6/ 19 (24.0)	7/ 23 (23.3)	0.954
Weight loss, n (%)	41/ 13 (75.9)	18/ 7 (72.0)	23/ 6 (79.3)	0.531
Weight loss, kg med (q1; q3)	10.0 (4.0; 15.0)	10.0 (0.7; 10.0)	10.0 (5.0; 15.0)	0.389
Diabetes mellitus, n (%)	24/ 33 (42.1)	14/ 12 (53.8)	10/ 21 (32.2)	0.100
Long-standing diabetes, n (%)	14/ 43 (23.3)	8/ 18 (30.8)	6/ 25 (19.4)	0.319
New-onset diabetes, n (%)	10/ 47 (16.6)	6/ 20 (23.1)	4/ 27 (12.9)	0.314
MIF, pg/mL med (q1; q3)	4.4 (2.6; 9.0)	4.1 (2.1; 7.9)	4.4 (2.9; 10.1)	0.212
MIF, cut off	29/ 31 (48.3)	13/ 11 (48.1)	16/ 17 (48.5)	0.979
IL-8/ CXCL8, pg/mL med (q1; q3)	7.5 (3.8; 35.5)	7.1 (2.7; 32.0)	9.7 (4.1; 36.4)	0.562
IL-8/ CXCL8 cut-off pg/mL	30/ 30 (50.0)	11/ 16 (40.7)	15/ 18 (45.5)	0.714
SCF, pg/mL med (q1; q3)	13.1 (9.5; 17.4)	9.5 (7.7; 11.9)	16.5 (14.2; 22.6)	0.000
SCF, cut-off pg/mL	33/ 27 (55.0)	-	-	0.000
Leucocytes/µL, med (q1; q3)	8.1 (6.6; 10.9)	7.7 (6.1; 11.2)	8.5 (6.8; 10.8)	0.625
Neutrophils/µL, med (q1; q3)	5.5 (4.4; 8.0)	5.0 (4.2; 7.9)	5.6 (4.9; 8.1)	0.211
Lymphocytes/µL, med (q1; q3)	1.6 (1.1; 2.1)	1.4 (1.1; 1.9)	1.7 (1.0; 2.1)	0.458
Hemoglobin g/dL, med (q1; q3)	12.4 (11.3; 13.7)	12.6 (11.7; 13.7)	12.3 (10.9; 13.8)	0.324
Thrombocytes/µL, med (q1; q3)	280.0 (205.5; 344.5)	255.0 (191.2; 323.0)	293.0 (236.0; 353.0)	0.451
ESR mm/h, med (q1; q3)	31.0 (20.7; 63.7)	66.0 (31.5; 113.0)	26.0 (20.0; 26.0)	0.052
CRP mg/L, med (q1; q3)	14.5 (4.9; 86.0)	17.4 (8.5; 135.2)	11.2 (3.0; 68.4)	0.138
Fibrinogen mg/dL med (q1; q3)	645.0 (539.0; 801.0)	797.0 (536.0; 902.5)	623.0 (537.7; 718.7)	0.149
Total bilirubin, mg/dL med (q1; q3)	1.4 (0.5; 7.4)	3.3 (0.6; 11.0)	1.2 (0.4; 6.4)	0.167
Direct bilirubin, mg/dL med (q1; q3)	0.7 (0.1; 5.0)	1.4 (0.1; 6.6)	0.4 (0.1; 4.5)	0.487
Creatinine, mg/dL med (q1; q3)	0.7 (0.6; 0.9)	0.6 (0.5; 0.7)	0.8 (0.7; 1.0)	0.010
Uric acid, mg/dL med (q1; q3)	4.4 (3.7; 5.7)	4.0 (3.6; 4.5)	5.0 (3.7; 6.3)	0.085
ASAT U/L, med (q1; q3)	54.0 (26.0; 140.0)	79.5 (32.2; 144.5)	32.5 (23.2; 133.5)	0.229

ALAT U/L, med (q1; q3)	56.5 (20.0; 186.5)	75.0 (31.0; 321.0)	44.0 (19.0; 157.0)	0.106
GGT U/L, med (q1; q3)	308.5 (38.5; 996.5)	324.0 (49.0; 965.0)	293.0 (35.0; 1064.0)	0.910
ALP U/L, med (q1; q3)	197.0 (82.5; 492.5)	276.5 (113.5; 459.5)	151.0 (70.0; 574.0)	0.358
Glycemia mg/dL, med (q1; q3)	120.0 (104.0; 144.0)	60.0 (36.5; 112.5)	57.5 (35.2; 82.7)	0.402
Amylase U/L, med (q1; q3)	60.0 (36.0; 93.5)	77.0 (16.5; 206.5)	32.0 (18.0; 86.5)	0.332
Lipase U/L, med (q1; q3)	40.5 (18.5; 105.5)	124.0 (108.0; 145.5)	113.0 (103.0; 141.0)	0.390
Total cholesterol mg/dL, med (q1; q3)	191.0 (122.7; 214)	192.0 (131.0; 241.0)	188.0 (118.5; 212.5)	0.689
Triglycerides mg/dL, med (q1; q3)	112.0 (94.0; 223.0)	123.0 (89.5; 286.5)	102.0 (91.0; 170.5)	0.651
Large tumor diameter, n (%)	25/ 22 (53.2)	12/ 9 (57.1)	13/ 13 (50.0)	0.626
Large tumor dimension, n (%)	14/ 23 (37.8)	6/ 10 (37.5)	8/ 13 (38.1)	0.970
Local tissular invasion, n (%)	40/ 6 (87.0)	19/ 2 (90.5)	21/ 4 (84.0)	0.516
Local vascular invasion, n (%)	29/ 15 (65.9)	13/ 7 (65.0)	16/ 8 (66.7)	0.908
Metastatic disease, n (%)	23/ 24 (48.9)	6/ 15 (28.6)	17/ 9 (65.4)	0.012
Fatigue, med (q1; q3)	34.0 (14.7; 40.7)	25.5 (10.7; 38.0)	36.0 (24.0; 41.5)	0.078

***** p-values were derived from the Mann–Whitney U test for continuous variables and the chi-square test for dichotomous ones. p-value < 0.05 was considered statistically significant.

Abbreviations: ALAT - alanine aminotransferase; ALP - alkaline phosphatase; ASAT - aspartate aminotransferase; CRP - C-reactive protein; ESR - erythrocyte sedimentation rate; HDL - high-density lipoprotein; GGT - gamma-glutamyl transferase; IL-8 - Interleukine-8; MIF - Macrophage migration inhibitory factor; SCF - Stem cell factor.

Table S4. Pancreatic cancer patients' characteristics stratified by the metastatic status

**Parameter**	**Cases** n=60	**without metastasis** n=24	**with metastasis** n=23	**p-value***
Gender, M/F (%M)	26/ 34 (43.3)	13/ 11 (54.2)	8/ 15 (34.8)	0.181
Age, years med (q1; q3)	69.5 (64.2; 76.0)	69.0 (57.2; 75.5)	70.0 (61.0; 76.0)	0.701
BMI, kg/m^2 med (q1; q3)	24.7 (21.9; 28.0)	23.4 (21.9; 25.9)	25.7 (21.5; 31.8)	0.286
Smoke, n (%)	16/ 41 (39.0)	8/ 16 (33.3)	5/ 15 (25.0)	0.390
Alcohol, n (%)	15/ 42 (26.3)	9/ 15 (37.5)	4/ 16 (20.0)	0.205
Meat-rich diet, n (%)	20/ 36 (35.7)	8/ 16 (33.3)	8/ 11 (42.1)	0.555
Sweet-rich diet, n (%)	16/ 41 (28.1)	8/ 16 (33.3)	3/ 17 (15.0)	0.162
High-fat diet, n (%)	13/ 44 (22.8)	8/ 16 (33.3)	1/ 19 (5.0)	0.020
Abdominal pain, n (%)	40/ 14 (74.1)	17/ 6 (70.8)	15/ 4 (78.9)	0.703
Nausea, n (%)	22/ 30 (42.3)	7/ 16 (30.4)	9/ 9 (50.0)	0.202
Diarrhea, n (%)	13/ 42 (23.6)	9/ 14 (39.1)	3/ 17 (15.0)	0.078
Weight loss, n (%)	41/ 13 (75.9)	17/ 6 (73.9)	14/ 5 (73.7)	0.987
Weight loss, kg med (q1; q3)	10.0 (4.0; 15.0)	10.0 (2.2; 11.2)	10.0 (4.0; 13.5)	0.833
Diabetes mellitus, n (%)	24/ 33 (42.1)	10/ 13 (43.5)	9/ 13 (40.9)	0.862
Long-standing diabetes, n (%)	14/ 43 (23.3)	5/ 18 (21.7)	6/ 16 (27.3)	0.666
New-onset diabetes, n (%)	10/ 47 (16.6)	5/ 18 (21.7)	3/ 19 (13.6)	0.477
MIF, pg/mL med (q1; q3)	4.4 (2.6; 9.0)	6.1 (2.7; 9.8)	4.0 (2.4; 8.0)	0.343
MIF, cut-off pg/mL	29/ 31 (48.3)	13/ 11 (54.2)	9/ 14 (39.1)	0.302
IL-8/ CXCL8, pg/mL med (q1; q3)	7.5 (3.8; 35.5)	14.1 (3.3; 43.1)	5.8 (4.1; 18.1)	0.170
IL-8/ CXCL8 cut-off pg/mL	30/ 30 (50.0)	14/ 10 (58.3)	7/ 16 (30.4)	0.054
SCF, pg/mL med (q1; q3)	13.1 (9.5; 17.4)	11.9 (8.5; 15.1)	15.4 (12.5; 19.9)	0.013
SCF, cut-off pg/mL	33/ 27 (55.0)	9/ 15 (37.5)	17/ 6 (73.9)	0.012
Leucocytes/µL, med (q1; q3)	8.1 (6.6; 10.9)	8.9 (7.1; 11.4)	8.3 (6.9; 11.5)	0.525
Neutrophils/µL, med (q1; q3)	5.5 (4.4; 8.0)	6.0 (4.5; 8.1)	5.6 (4.8; 7.6)	0.856
Lymphocytes/µL, med (q1; q3)	1.6 (1.1; 2.1)	1.4 (1.0; 2.2)	1.6 (0.9; 2.1)	0.955
Hemoglobin g/dL, med (q1; q3)	12.4 (11.3; 13.7)	12.6 (11.4; 14.0)	12.2 (10.4; 13.2)	0.203
Thrombocytes/µL, med (q1; q3)	280.0 (205.5; 344.5)	301.0 (208.0; 326.0)	293.5 (191.2; 353.0)	0.973
ESR mm/h, med (q1; q3)	31.0 (20.7; 63.7)	66.5 (30.5; 112.7)	23.0 (18.5; 32.0)	0.014
CRP mg/L, med (q1; q3)	14.5 (4.9; 86.0)	20.1 (5.7; 139.2)	14.0 (2.5; 83.9)	0.411
Fibrinogen mg/dL, med (q1; q3)	645.0 (539.0; 801.0)	797.0 (645.0; 901.0)	557.0 (519.0; 703.0)	0.013
Total bilirubin, mg/dL med (q1; q3)	1.4 (0.5; 7.4)	2.2 (0.5; 11.1)	0.9 (0.4; 3.0)	0.262
Direct bilirubin, mg/dL med (q1; q3)	0.7 (0.1; 5.0)	1.5 (0.1; 6.7)	0.2 (0.1; 1.5)	0.171
Creatinine, mg/dL med (q1; q3)	0.7 (0.6; 0.9)	0.7 (0.6; 0.9)	0.8 (0.6; 1.0)	0.465
Uric acid, mg/dL med (q1; q3)	4.4 (3.7; 5.7)	4.0 (3.4; 4.9)	5.9 (3.7; 8.1)	0.040
Glycemia, mg/dL med (q1; q3)	54.0 (26.0; 140.0)	129.0 (108.0; 147.0)	120.0 (103.5; 126.0)	0.279
ASAT U/L, med (q1; q3)	56.5 (20.0; 186.5)	62.5 (27.7; 144.5)	32.5 (19.2; 68.2)	0.117
ALAT U/L, med (q1; q3)	308.5 (38.5; 996.5)	68.0 (44.0; 345.0)	39.0 (16.2; 62.0)	0.014
GGT U/L, med (q1; q3)	280.0 (205.5; 344.5)	3950 (58.0; 886.0)	138.0 (35.0; 1007.0)	0.448

ALP U/L, med (q1; q3)	197.0 (82.5; 492.5)	217.0 (137.0; 378.0)	103.0 (76.0; 375.7)	0.284
Glycemia mg/dL, med (q1; q3)	120.0 (104.0; 144.0)	129.0 (108.0; 147.0)	120.0 (103.5; 126.0)	0.279
Amylase U/L, med (q1; q3)	60.0 (36.0; 93.5)	64.0 (35.7; 111.2)	57.5 (35.2; 82.7)	0.501
Lipase U/L, med (q1; q3)	40.5 (18.5; 105.5)	38.0 (8.0; 125.0)	31.0 (18.0; 70.0)	0.849
Total chol mg/dL, med (q1; q3)	191.0 (122.7; 214)	197.5 (165.2; 208.5)	163.5 (113.0; 223.7)	0.375
Triglycerides mg/dL, med (q1; q3)	112.0 (94.0; 223.0)	186.0 (99.7; 287.5)	105.0 (84.2; 152.7)	0.172
Large tumor diameter, n (%)	25/ 22 (53.2)	8/ 13 (38.1)	11/ 9 (55.0)	0.636
Large tumor dimension, n (%)	14/ 23 (37.8)	6/ 11 (35.3)	6/ 11 (35.3)	0.303
Local tissular invasion, n (%)	40/ 6 (87.0)	19/ 5 (79.2)	17/17 (100)	0.045
Local vascular invasion, n (%)	29/ 15 (65.9)	14/ 10 (58.3)	13/ 4 (76.5)	0.228
Metastatic disease, n (%)	23/ 24 (48.9)	-	-	0.000
Fatigue, med (q1; q3)	34.0 (14.7; 40.7)	36.0 (12.5; 44.7)	34.0 (15.5; 39.0)	0.362

***** p-values were derived from the Mann–Whitney U test for continuous variables and the chi-square test for dichotomous ones. p-value < 0.05 was considered statistically significant.

Abbreviations: ALAT - alanine aminotransferase; ALP - alkaline phosphatase; ASAT - aspartate aminotransferase; CRP - C-reactive protein; ESR - erythrocyte sedimentation rate; HDL - high-density lipoprotein; GGT - gamma-glutamyl transferase; IL-8 - Interleukine-8; MIF - Macrophage migration inhibitory factor; SCF - Stem cell factor;
